# Alterations in function and expression of ABC transporters at blood-brain barrier under diabetes and the clinical significances

**DOI:** 10.3389/fphar.2014.00273

**Published:** 2014-12-10

**Authors:** Li Liu, Xiao-Dong Liu

**Affiliations:** Center of Drug Metabolism and Pharmacokinetics, College of Pharmacy, China Pharmaceutical UniversityNanjing, China

**Keywords:** blood-brain barrier, ABC transporters, diabetes, Alzheimer's disease, amyloid β-peptide, ABCB1, ABCG2

## Abstract

Diabetes is a systematic metabolic disease, which often develops a number of well-recognized vascular complications including brain complications which may partly result from the dysfunction of blood-brain barrier (BBB). BBB is generally considered as a mechanism for protecting the brain from unwanted actions resulting from substances in the blood and maintaining brain homeostasis via monitoring the entry or efflux of compounds. ATP-binding cassette (ABC) family of transporters including P-glycoprotein (P-GP) and breast cancer-related protein (BCRP), widely expressed in the luminal membrane of the microvessel endothelium and in the apical membrane of the choroids plexus epithelium, play important roles in the function of BBB. However, these transporters are easily altered by some diseases. The present article was focused on the alteration in expression and function of both P-GP and BCRP at BBB by diabetes and the clinical significances.

## Introduction

The blood-brain barrier (BBB) is a multicellular vascular structure that separates the central nervous system (CNS) from the peripheral blood circulation. The basic structural and functional unit of the BBB is considered to be the so-called neurovascular unit, which is composed of at least four cell types: brain capillary endothelial cells comprise the brain capillaries of the cerebral vasculature; pericytes sit on top of endothelial cells sharing the same basal membrane; astrocytes surround the brain capillaries and cover them with their endfeet; and neurons directly innervate the microvasculature. Together these cells of the neurovascular unit control barrier function. By tightly controlling the passage of molecules and ions, instantaneously delivering nutrients and oxygen according to current neuronal needs and protecting the brain from toxins and pathogens, the BBB maintains an environment that allows neurons to function properly (Obermeier et al., 2013).

Beyond barrier function, influx and efflux are actively regulated at the blood-brain interface. At the molecular level, several factors contribute to the unique functional role of the BBB. Tight junctions in the capillary endothelium seal the spaces between neighboring endothelial cells and represent a passive, physical barrier that restricts paracellular diffusion of solutes from blood to brain. To meet the high nutrient and energy demand of the brain, influx transporters facilitate brain uptake of glucose, amino acids, ions, and other nutrients from the blood. ATP-binding cassette (ABC) efflux transporters on the other hand extrude metabolic wastes into the blood and form a selective, active barrier protecting the CNS by limiting xenobiotics, including toxins and a large number of drugs, from entering the brain (Hartz and Bauer, 2011). Thus, the BBB is a complex and fine-tuned transport machine that balances influx of nutrients and efflux of wastes, toxins, and drugs to maintain CNS homeostasis. The ABC efflux proteins of this transport machinery are critical for the functional barrier and present a formidable impediment to brain delivery of therapeutic drugs.

ABC transporters belong to one of the largest and most ancient protein superfamilies and are highly conserved between species and throughout evolution. ABC transporters are transmembrane proteins that transport lipids, sterols, metabolic wastes, and therapeutic drugs across intra- and extracellular membranes. These transport processes are ATP-driven (active transport) and can, therefore, be directed against a solute's concentration gradient. By now, the ABC transporter superfamily comprises 49 human proteins divided into seven subfamilies that have been designated ABCA to ABCG (Pahnke et al., 2014). They are expressed in every cell type of the brain and mediate the transport of a wide variety of substances.

For vertebrates, three ABC subfamilies, B, C, and G, contain transporters that function as multispecific, ATP-driven efflux pumps, and largely handle foreign chemicals (xenobiotics). As a rule, these ABC transporters are expressed in all cells, but they are most highly expressed in barrier (such as the BBB) and excretory tissues. Certainly, for an efflux transporter to be effective in limiting blood to brain movement of drugs and neurotoxicants and driving efflux of potentially toxic metabolites, it should be localized to the luminal plasma membrane. Functional studies with wild-type and knock-out rodents as well as immunohistochemistry indicate that luminal membrane localization is certain for P-glycoprotein (ABCB1), Multidrug Resistance-Associated Protein 2, MRP2 (ABCC2), and breast cancer resistance protein (ABCG2) (BCRP). They have the potential to affect many signaling molecules, waste products of normal metabolism, therapeutic drugs, environmental toxicants, and toxicant metabolites efflux out of the CNS (Miller, 2014).

Under the physiological state, BBB is generally considered as a mechanism for protecting the brain from unwanted actions resulting from substances in the blood and maintaining brain homeostasis via monitoring the entry or efflux of compounds. The BBB is not a static anatomical boundary, but a dynamic interface capable of rapid response to stressors including hypoxia, inflammation, trauma, and pain. In a number of brain pathologies, loss of BBB function and changes in ABC transporter expression have been suggested as a prognostic factor in the progression of these diseases (Wanek et al., 2013). Furthermore, the BBB and ABC transporter dysfunction exacerbates numerous diseases and pathologies including stroke, Alzheimer disease, Parkinson disease, diabetes, acute liver failure, etc. Loss of BBB integrity (i.e., leak or dysfunction of ABC transporters) exposes the brain to potentially harmful concentrations of substances in the peripheral circulation (e.g., ions, amino acids, neurotransmitters, proteins, and other macromolecules) that may disrupt brain homeostasis and adversely affect neuronal signaling (Abbott et al., 2010).

Diabetes mellitus (DM) is a systematic metabolic disease, which develops a number of well-recognized macro- and microvascular complications related to endothelial dysfunction (Stumvoll et al., 2005). Diabetic macrovascular complications involve vessel obstructions, such as coronary artery diseases, atherosclerosis, and peripheral vascular diseases. Microvascular pathologies include retinopathy, nephropathy, and neuropathy. Direct damage to small blood vessels, particularly by hyperglycemia, is manifested by endothelial dysfunction, diminished perfusion, abnormal endothelial cell proliferation, and increased vessels permeability. Type 2 DM patients exhibit similar microvascular damage within the CNS which may result in increased incidence of cognitive deterioration, vascular dementia, lacunar infarcts, hemorrhages and Alzheimer's disease (AD) (Serlin et al., 2011). These diabetes induced brain microvascular complications have disgusting consequences in dysfunction of the BBB. Several phenomena known to disrupt the BBB integrity, such as transient cerebral ischemia, hypertension and hyperosmolality, are commonly associated with DM. DM is also associated with changes in ABC transport functions in the cerebral microvessels. Under diabetic conditions, the integrity of BBB is compromised and molecules that are normally confined to the blood may enter the parenchyma leading to dramatic changes in brain structure and function (Alves et al., 2012). The diabetes-induced perturbations to cerebral microvessels may disrupt homeostasis and contribute to long-term cognitive and functional deficits of the CNS. The present article summarizes and highlights the accumulating evidence in the literature which describe the role of altered expression and function of both P-GP and BCRP in the pathogenesis of diabetes and the clinical significances.

## Alteration in BBB permeability by diabetes

### Altered blood-brain barrier structure in diabetes mellitus

Alteration of cerebral microvessel structure, notably decreased capillary density and disruption of tight junction function, is one of the important reasons that diabetes disrupts BBB function. Although BBB permeability under diabetic status has been widely investigated, the results are often controversial. Dai et al. reported that no obvious differences in the staining pattern of IgG and albumin were observed between brain samples of persons with diabetes and controls (Dai et al., 2002). But Starr's study showed the increased BBB permeability to gadolinium diethylenetriamine pentaacetic acid in patients with type 2 diabetes using gadolinium magnetic resonance imaging (Starr et al., 2003). Antibodies against serum S100B and NSE (CNS proteins) were found to be significantly increased in both type 1 and type 2 diabetic subjects compared to controls, implying that diabetes in humans may be associated with alterations in the integrity of the BBB (Hovsepyan et al., 2004). Animal experiment demonstrated that diabetes significantly reduced cerebral tight junction protein occludin content, but another tight junction protein zonula occludens-one content was not significantly altered, indicating that diabetes alters the molecular anatomy of the tight junctions in cerebral tissue by altering the content of select structural proteins (Chehade et al., 2002).

### Metabolic changes on BBB in diabetes mellitus

Several studies have exhibited alterations in some nutrient transporters at BBB under diabetic status. In hyperglycemic non-obese diabetic mice, diabetes down-regulated both BBB permeability to glucose and transporter maximal velocity of glucose without alteration in the half-saturation constant (Cornford et al., 1995), which may be attributed to the downregulated glucose transporters (Hou et al., 2007). The decrease in expression of glucose transporters at BBB impairs glucose transport into the brain, leading to mental retardation (De Vivo et al., 1991) although there was a report showing transport of glucose across BBB was not altered in poorly controlled type 1 diabetes (Fanelli et al., 1998). Influx of vitamin C to the brain is also mediated via the glucose transporter, indicating that the decreased glucose transporter may be responsible for depleted brain content of vitamin C in diabetic animals (Minamizono et al., 2006). In addition, diabetes decreased monocarboxylic acid transporter expression, leading to increase in BBB permeability to acetate acid (Mason et al., 2006). Insulin uptake by brain was reported to increase under diabetic status, however, this increase was not due to acute changes in the serum levels of glucose or insulin, altered vascular space, or catabolic events (Banks et al., 1997).

### Mechanisms of BBB breakdown in diabetes mellitus

The underlying molecular changes leading to BBB dysfunction under diabetic condition are not completely clear, but may involve increased expression of matrix metalloproteases (MMP), confused ketone body levels and inflammation.

MMP activity is often increased in diabetic patients, indicating that higher plasma MMP activity increased BBB permeability in diabetic condition via degradation of tight junction proteins. Animal study (Hawkins et al., 2007a) showed that increase of BBB permeability to sucrose in streptozotocin-treated rats was associated with the decreased level of tight junction proteins occludin and ZO-1 and the increase of MMP activity in plasma, which verified above deduction. Insulin treatment may attenuate the BBB hyperpermeability to sucrose.

In diabetic ketoacidosis, the relationship between ketone bodies and brain metabolism is well-documented (Roe et al., 1996). Two ketone bodies β-hydroxybutyrate and acetoacetate cross BBB via a monocarboxylic acid transport system. This transport was increased by elevated blood concentration of ketone bodies. β-hydroxybutyrate and acetoacetate increased BBB permeability and induced brain edema via increasing the production of vascular endothelial growth factor and vasoconstrictor endothelin-1 (Isales et al., 1999).

Inflammatory mechanisms underlying vascular pathology in DM are possibly common to the vasculature in the periphery and CNS. Formation of advanced glycation end products (AGEs) via glycation of blood proteins is a consequence of hyperglycemia, and it results in decreased kidney function and small vessels pathology. AGEs accumulation may induce vascular inflammation by the interactions between AGEs and AGE-specific receptors (RAGE) (Meerwaldt et al., 2009). AGEs activation of endothelial RAGE promotes upregulation of endothelial adhesion molecules including vascular cell adhesion molecule 1 (VCAM-1) and activates transcription factor nuclear factor-κB (NF-κB). The former increases monocyte adhesiveness and vascular permeability while the latter regulates multiple proinflammatory and proatherosclerotic target genes in endothelial and vascular smooth muscle cells as well as in macrophages (Piga et al., 2007).

## ABC transporters at BBB and diabetes

### P-GP and diabetes

Diabetes is often associated with disorder of energy metabolism and increases of pro-inflammatory cytokines in the systemic circulation, which inferred that diabetes may alter expression and function of ABC transporters at BBB, in turn, affect BBB permeability. We once reported that diabetes down-regulated P-GP expression at BBB of diabetic rats, leading to increase in brain distribution of vincristine and rhodamine (Liu et al., 2006). Seventy two hour exposure to serum of diabetic rats down-regulated function and protein levels of P-GP in primarily cultured rat brain microvessel endothelial cells (rBMECs), which may be reversed by adding of insulin (Liu et al., 2008a). We also found that the effects of diabetes on Abcb1 mRNA levels was dependent on both brain regions and Abcb1 species in streptozotocin (STZ)-induced diabetic rats. Diabetes clearly down-regulated Abcb1a mRNA levels in the cerebral cortex of diabetic rats, while up-regulated Abcb1a mRNA levels in hippocampus. It was contrast to finding in Abcb1a mRNA that the levels of Abcb1b mRNA cerebral cortex of diabetic rats were increased rather than decreased. The Abcb1b mRNA levels were not significantly affected by diabetes. The increased P-GP proteins were also observed in hippocampus of diabetic rats (Zhang et al., 2011). Similarly, expression of P-GP in brain striatum of type 2 diabetic mice was increased (Wu et al., 2009). *In vivo* study showed that insulin treatment not only restored the impaired function and expression of P-GP at BBB by diabetes, but also upregulated expression and function of P-GP at BBB of normal rats (Liu et al., 2008a). In rBMECs, insulin dose-dependently increased expression and function of P-GP, which may be abolished by insulin receptor antibody (Liu et al., 2009). Above results give a clue that low level of insulin in plasma is a main factor resulting in impairment of P-GP expression at BBB. Further investigation showed that insulin up-regulated P-GP expression and function at BBB via activating PKC/NF-κ B pathway (Liu et al., 2009). NO is also considered to regulate expression and function of P-GP, but our previous study on Caco-2 cells showed that the effects of NO donors on P-GP function and expression were dependent on exposure time. Four hour exposure to NO donors impaired P-gp function and expression, whereas 24-h exposure stimulated P-gp function and expression (Duan et al., 2012). Other factors such as inflammatory cytokines and related molecules (von Wedel-Parlow et al., 2009; Poller et al., 2010) may be involved in expression and function of P-GP at BBB. Several studies have showed that cytokines exert dose-and time-dependent modulation of efflux transporters. In RBE4 cells, tumor necrosis factor-alpha (TNF-α) upregulated P-GP expression, accompanied by decrease in cellular uptake of the P-GP substrate vinblastine (Yu et al., 2007). In isolated rat brain capillaries, TNF-α shows biphasic effects on the expression and activity of P-GP with low dose decreasing but longer exposure (6 h) increasing the expression and activity of P-GP. This is probably explained by differences in the signaling pathways activated (Bauer et al., 2007). Following 72-h exposure to TNF-α, human endothelial cells showed an increase of P-GP expression, but P-GP activity was unaltered (Poller et al., 2010). In primary cultures of porcine brain capillary endothelial cells, P-GP protein expression was transiently increased after TNF-α addition within 6 h of incubation followed by a reduction after 24 and 48 h, whereas the Abcb1 mRNA levels were not changed. It was also found that Interleukin-1β (IL-1β) decreased the expression and activity of P-GP (von Wedel-Parlow et al., 2009).

Absence of functional P-GP at BBB leads to highly increased brain penetration of a number of important drugs, inducing dramatically increased neurotoxicity, or fundamentally altered pharmacological effects of the drugs on CNS. Phenobarbital, an antiepileptic and sedative drug, was shown to be a substrate for P-GP (Yang and Liu, 2008). The impaired function and expression of P-GP on BBB of diabetic mice resulted in increased brain distribution and pharmacological effect of phenobarbital (Liu et al., 2007a). Kamei et al. also reported that the systemic administration of second-generation H1-receptor antagonists (epinastine and cetirizine) showed CNS depressant effects in STZ-induced diabetic mice, accompanied by lower P-GP expression levels in the whole brain (Kamei et al., 2005).

### BCRP and diabetes

BCRP, another ABC transporter was reported to be downregulated at BBB of diabetic rats, accompanied by increases in brain distribution of prazosine and cimetidine (Liu et al., 2007b). Insulin treatment may attenuate the impairment of Bcrp expression and function induced by diabetes. Neither aminoguanidine (an inhibitor of advanced glycation end product) nor metformin treatment prevented the impairment of Bcrp function and expression in brain cortex of diabetic rats although aminoguanidine and metformin levels of advanced glycation end product and glucose in plasma diabetic rats were significantly suppressed by aminoguanidine and metformin respectively, indicating contribution of insulin to downregulation of BCRP. However, in rBMECs, we found that unlike P-GP, insulin itself suppressed rather than increased expression and function of Bcrp (Liu et al., 2011), indicating existence of other factors impairing expression and function of BCRP at BBB. von Wedel-Parlow et al. reported that TNF-α and IL-1β rapidly decreased Abcg2 mRNA expression within 6 h. Long-term treatment with the vasoconstrictor endothelin-1 (TNF-α downstream agent) induced Abcg2 protein expression. IL-1β caused a continuous decrease in protein expression of BCRP (von Wedel-Parlow et al., 2009). In immortalized brain endothelial cells (hCMEC/D3), IL-1β, IL-6 and TNF-α significantly reduced levels of BCRP mRNA. IL-1β showed the strongest inhibitory effect on expression of BCRP protein among the three proinflammatory cytokines. BCRP activity was also significantly inhibited by IL-1β, IL-6 and TNF-α, assessed by mitoxantrone uptake experiments (Poller et al., 2010).

### ABC transporters, diabetes and alzheimer's disease

#### ABC transporters mediate transport of Aβ across BBB

**Figure 1:**
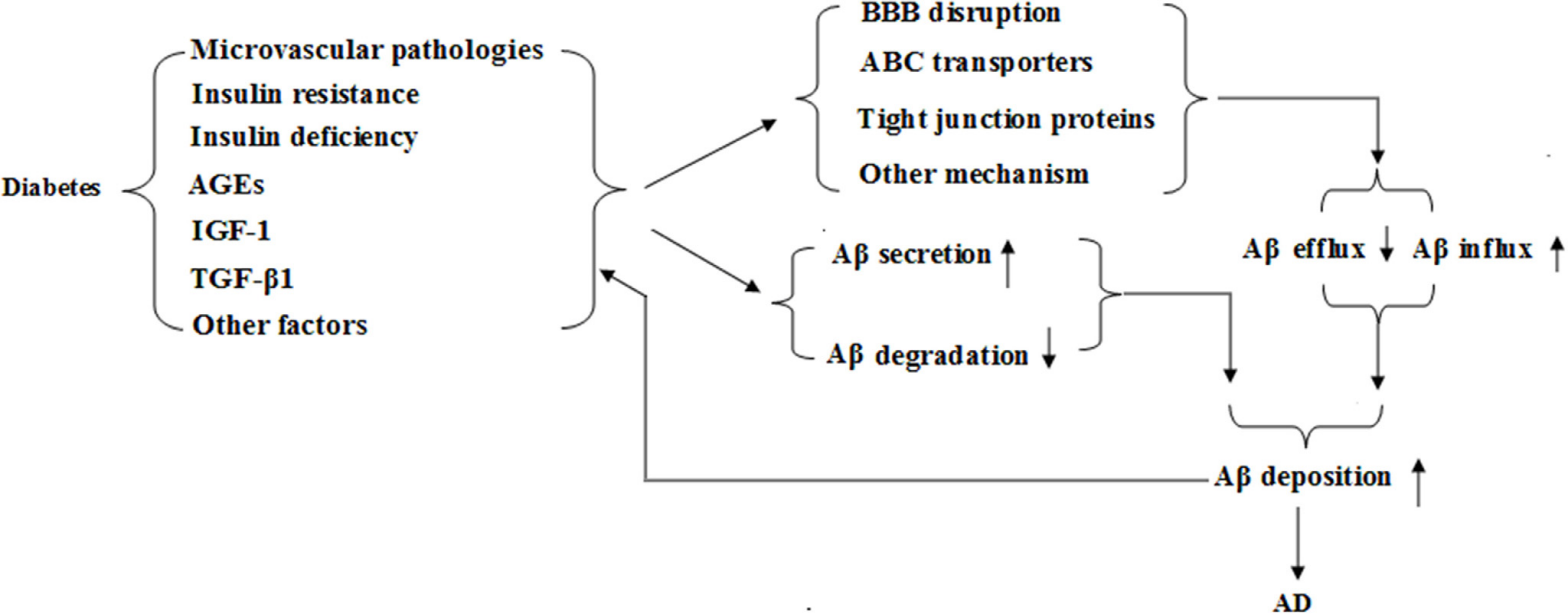
**Possible relationships in diabetes, blood brain barrier (BBB) function, β-amyloid and Alzheimer's disease (AD)**.

Recent studies have revealed that DM is a risk factor for cognitive dysfunction or dementia, especially those related to AD. AD is characterized by generation and deposition of amyloid β-peptide (Aβ) within extracellular spaces of the brain, which is believed to be the key player in the pathogenesis of AD (Vogelgesang et al., 2002). Animal and clinical reports showed that diabetes increased accumulation of Aβ in brain (Liu et al., 2008b; Alafuzoff et al., 2009; Tomita et al., 2013) although roles of Aβ in AD progression with diabetes need further verification. Possible relationships in diabetes, BBB function, Aβ, and AD were shown in Figure 1.

Several studies (Kuhnke et al., 2007; Jedlitschky et al., 2010) have showed that P-GP (ABCB1) mediated transport of Aβ across BBB. Our previous study demonstrated that diabetes increased Aβ level in hippocampus and cortex of rats. Insulin treatment may restore the increase of Aβ level induced by diabetes. Further study showed that the increase may partly come from both decrease in efflux of Aβ from brain to blood and increase in influx of Aβ from blood to brain, which were in line with impairment of P-GP function and expression (Liu et al., 2008b). This finding infers that increased accumulation of brain Aβ induced by diabetes at least is attributed to impaired expression and function of P-GP at BBB, which seems to explain that diabetic condition contributes to the pathogenesis of AD (Liu et al., 2008b). In a clinical trial with patients with mild to moderate AD using rifampicin, a potent inducer of P-gp, a reduced cognitive decline was observed after 12 months of treatment, presumably because the drug was able to improve the clearance of Aβ from the brain via the enhancement of P-gp mediated transport (Loeb et al., 2004). It was interesting that Aβ itself was reported to down-regulate expression of P-GP in brain and dosing of the animals with the PXR ligand pregnenolone-16 alpha-carbonitrile upregulated P-gp expression and reduced cerebral β-amyloid deposition (Hartz et al., 2010), inferring that accumulation of Aβ in turn further downregulated P-GP expression at BBB.

BCRP also mediate transport of endogenous substances across BBB. Several reports (Tai et al., 2009; Xiong et al., 2009; Do et al., 2012) have verified that BCRP mediate transport of Aβ across BBB. The role of ABCG2 in efflux of Aβ across BBB was investigated in Abcg2-null mice after intravenous injection of labeled Aβ (Xiong et al., 2009; Shen et al., 2010; Zhang et al., 2013). The results demonstrated that Abcg2-null mice significantly accumulated more Aβ in their brains than wild-type mice. *In situ* brain perfusion technique, GF120918 (dual inhibitor of Abcb1 and Abcg2) strongly enhanced the uptake of [3H] Aβ 1-40 by the brains of Abcb1-deficient mice, but not by the brains of Abcb1/Abcg2-deficient mice (Do et al., 2012). This role was further confirmed by *in vitro* studies utilizing ABCG2 overexpressing cells and hCMEC/D3 (Tai et al., 2009; Xiong et al., 2009). Above results give a concept that BCRP and P-GP “team up and work together” in transporting chemotherapeutics at BBB may possibly be extended to their handling of Aβ (Wolf et al., 2012). Unlike P-GP, Xiong and colleagues reported that ABCG2 gene was significantly up-regulated in the brains of AD patients and AD animals (Xiong et al., 2009), however, this induction was not copied by *in vitro* study on hCMEC/D3 (Xiong et al., 2009; Kania et al., 2011). The up-regulation of ABCG2 in AD brain was considered to be an adaptive response to relieve oxidative stress and protect brain cells/tissue against ROS induced damage and inflammatory response via inhibiting NF-κB signaling pathway (Shen et al., 2010). ABCG2 was also reported to plays a protective role against oxidative stress and anti-inflammation (Shen et al., 2010; Zeng et al., 2012). A study with HEK293 showed that transfection with ABCG2 significantly inhibited ROS formation by both tert-Butyl hydroperoxide and H_2_O_2_, indicating that ABCG2 decreased oxidative capacity of HEK293 cells or/and increased antioxidant capacity of the cells. Further investigation showed that over-expression of ABCG2 decreased H_2_O_2_-induced cell death, accompanied by decrease in IL-8 secretion and inhibition of NF-kB activation. In the brains of Abcg2-knockout mice, the increased Aβ accumulation was in line with NF-κ B activation. In Neural-derived mouse neuroblastoma cell line 2a-695 cells (expresses APP and produces β-amyloid peptides), transfection with ABCG2 significantly decreased secretion of Aβ1-40 peptides and downregulated activities of α-, β, and γ- secretases in cells (Shen et al., 2010). In Tg-SwDI/Abcg2-KO mice, Abcg2 deficiency increases oxidative stress and alters inflammatory response in the brain and exacerbates cognitive/memory deficit compared with Tg-SwDI mice, wild type mice and Abcg2-KO mice, accompanied by decrease in levels of total brain GSH and increase in levels of lipid/DNA oxidation (Zeng et al., 2012). All these results show important roles of BCRP in accumulation of Aβ in brain of diabetic patients and animals.

ABCB1 and ABCG2 are not the only ABC transporters found to be associated with Aβ transport. Two further ABC transporters, ABCA7 and ABCC1, appeared on the stage of AD. In AD patients ABCA7 expression was found to be higher than in non-demented people (Vasquez et al., 2013). Kim et al. evaluated the effect of ABCA7 on Aβ pathology using ABCA7 knockout mice that were crossed to the J20 AD model. They found a robust increase of insoluble Aβ and plaque load in the brains of ABCA7-deficient mice, but were unable to fully elucidate the mechanism behind this observation. Using bone marrow derived macrophages they showed a decreased ability of ABCA7 knockout cells to phagocytose oligomeric Aβ, pointing toward a possible mechanism of action (Kim et al., 2013). ABCC1 was also found to influence Aβ brain burden. An up to 14-fold increase of Aβ42 in ABCC1 knock-out mice is the so far greatest impact of an ABC transporter in AD mouse models. Moreover, amyloid burden was reduced by up to 80% in APPPS1 mice via functional activation of ABCC1 (Krohn et al., 2011). ABCC1 is expressed in capillary endothelia of the blood–brain barrier, in neural stem and progenitor cells (Schumacher et al., 2012). Its function is regulated by mitochondria and influenced by mitochondrial polymorphisms (Scheffler et al., 2012). However, it is also different to the other transporters because one important site of high expression in the brain is the choroid plexus.

#### Insulin/IGF-1 and TGF-β1 are involved in Aβ homeostasis

Insulin resistance, which is often associated with type 2 DM, may induce a deficiency of insulin effects in the CNS. Some research suggests that insulin resistance accelerates Alzheimer-related pathology through its effects on Aβ metabolism. Recent data also suggest that brains of patients with AD are insulin and insulin-like growth factor-1 (IGF-1) resistant. β-amyloid oligomers are responsible for downregulation of neuronal insulin receptors, whereas Aβ monomers are able to activate insulin/IGF-1 receptor signaling. It has been hypothesized that depletion of β-amyloid monomers, occurring in the preclinical phase of AD, might be the cause of early insulin/IGF-1 signaling disturbances that anticipate cognitive decline (Giuffrida et al., 2012).

Insulin directly increases Aβ secretion from neurons by accelerating peptide trafficking to the plasma membrane (Gasparini et al., 2001) and promotes Aβ degradation by regulating the expression of the insulin-degrading enzyme, a metalloprotease that catabolizes both insulin and Aβ (Zhao et al., 2004). IGF-1 increases Aβ clearance from the brain by enhancing transport of Aβ-carrier proteins (e.g., albumin and transthyretin) into the brain (Carro et al., 2002). Hence, insulin and IGF-1 seem to act in conjunction as regulators of brain Aβ content, and systemic conditions altering their interplay could indirectly promote Aβ oligomerization. For example, aging (the main risk factor for AD) is associated with low serum levels of IGF-1 (Piriz et al., 2011), and type 2 diabetes is associated with peripheral hyperinsulinemia and low brain insulin levels that could result in reduced Aβ clearance (Giuffrida et al., 2012). Insulin and IGF-1 regulate multicargotransporters influencing trafficking of several molecules including Aβ from the brain to the blood as well as to the cerebrospinal fluid (CSF) and possibly vice versa. Furthermore, insulin and related peptides regulate neurovascular coupling changing regional blood flow. Thus, positive effects of peripheral insulin/IGF-1 administration on AD pathology might be due to changes in the BBB and/or in the transport between the CSF/blood and the brain. Haploinsufficiency of the IGF-1 receptor (IGF-1R) (IGF-1R+/- mice) as well as neuronal deficiency of the insulin receptor (IR) (nIR-/- mice) or IGF-1R (nIGF-1R-/- mice) leads to delayed Aβ accumulation when crossed with mouse models for AD. Furthermore, insulin receptor substrate (IRS)-2 knockout mice (IRS-2-/- mice) show reduced Aβ levels in an Alzheimer background. These data suggest beneficial effects of decreased neuronal insulin/IGF-1 signaling on Alzheimer-pathology and question the therapeutic outcome of long-term administration of insulin or IGF-1 in patients with AD (Zemva and Schubert, 2014).

TGF-β1 is well-known to modulate blood vessel development and maturation. Oligodendrocyte precursor cells (OPCs) are an important source of TGF-β1 that supports BBB integrity during development. OPCs derived TGF-β1 activates the MEK/ERK pathway in cerebral endothelium via TGF-β receptor, and the signaling cascade eventually increases the expression levels of tight-junction proteins to promote the BBB integrity (Seo et al., 2014). Reducing neuronal TGF-β1 signaling in mice resulted in age-dependent neurodegeneration and promoted Aβ accumulation and dendritic loss in a mouse model of AD (Wyss-Coray et al., 2001). In cultured cells, reduced TGF-β signaling caused neuronal degeneration and resulted in increased levels of secreted Aβ and β-secretase-cleaved soluble amyloid precursor protein (Tesseur et al., 2006). Hence, reduced neuronal TGF-β1 signaling increases age dependent neurodegeneration and AD-like symptoms. Improvement of TGF-β1 signaling may be a novel therapeutic approach to AD, simultaneously targeting a neurodegenerative pathway and preventing Aβ deposition (Caraci et al., 2012). A recent study reported that TGF-β1 potently regulates P-GP activity and ABCB1 mRNA at the developing BBB and both ALK5 and ALK1 pathways are implicated in the regulation of P-GP function (Baello et al., 2014). The research indicates that aberrations in TGF-β1 levels at the BBB may lead to substantial changes in brain exposure to P-GP substrates including Aβ, triggering consequences for neurodegenerative disease. It is consistent with the above findings which illustrate that neuronal TGF-β1 signaling has been impaired in AD, resulting in impaired function and expression of P-GP at the BBB.

However, the regulation of BBB by TGF-β1 is contradictory in DM. It is generally accepted advanced glycation endproducts (AGEs) are considered to be an important factor in the formation of diabetic vascular complications. Takeshita et al. reported that AGEs decreases in the expression of proteins comprising tight junctions through the production of TGF-β1 in the BBB (Takeshita et al., 2014). This finding suggests high level of AGEs activate brain TGF-β1 signal pathway, leading to disturbance of BBB under diabetic condition. Clinical and animal study also showed that elevated plasma and urinary levels of TGF-β1 in diabetic patients and inhibition of TGF-β1 action is beneficial for diabetic nephropathy (Li and Hölscher, 2007).

## Future perspectives

ABC transporters including P-GP, BCRP and MRPs at BBB play an important role in the transporting compounds across BBB. The deliberate modulating ABC-transporters may mediate the permeability of their substrates including drugs and endogenous substances across BBB, affecting activity/toxicity of their substrates on CNS. Based on current knowledge, we propose that P-GP and BCRP are able to contribute to brain complications under diabetic conditions. However, we remain far from the exact contribution these transporters have to the disease. For example, how diabetes affect ABC transporters in brain is still unclear. Thus, a better understanding of ABC transporters functions in the human brain is of major pharmacological importance to the development and optimization of therapeutic strategies that target these transporters.

Here, we only discussed alterations in expression and function of P-GP and BCRP at BBB under diabetic status. In fact, other transporters such as MRP2 (Hawkins et al., 2007b) and ABCA1 (Wang et al., 2012) were also investigated in brain of diabetic rats, but the clinical significances needs further investigation. In addition, it should be mentioned that effects of diabetes on expression and function of ABC transporters at BBB are often dependent on species of ABC transporters, brain regions, duration of diabetes and diabetic type, which may gave different results.

### Conflict of interest statement

The authors declare that the research was conducted in the absence of any commercial or financial relationships that could be construed as a potential conflict of interest.
